# Prehospital transportation to therapeutic hypothermia centers and survival from out-of-hospital cardiac arrest

**DOI:** 10.1186/s12913-015-1199-z

**Published:** 2015-12-02

**Authors:** Derek DeLia, Henry E. Wang, Jared Kutzin, Mark Merlin, Jose Nova, Kristen Lloyd, Joel C. Cantor

**Affiliations:** Center for State Health Policy, Rutgers University, 112 Paterson St., Room 540, New Brunswick, NJ 08901 USA; Department of Emergency Medicine, University of Alabama at Birmingham, 266N Jefferson Tower, 625 19th Street south, Birmingham, AL 35249-7013 USA; Simulation Center at Winthrop University Hospital, Englewood Hospital and Medical Center, Winthrop University Hospital, 259 First St, Mineola, NY 11501 USA; Rutgers School of Public Health, Attending, Emergency Medicine, Newark Beth Israel Medical Center, Newark Beth Israel Medical Center, 201 Lyons Avenue, Newark, NJ 07112 USA

**Keywords:** Cardiac arrest, Treatment outcomes, Emergency care, Prehospital emergency medical services (EMS), Instrumental variables

## Abstract

**Background:**

Clinical trials supporting the use of therapeutic hypothermia (TH) in the treatment of out-of-hospital cardiac arrest (OHCA) are based on small patient samples and do not reflect the wide variation in patient selection, cooling methods, and other elements of post-arrest care that are used in everyday practice. This study provides a real world evaluation of the effectiveness of post-arrest care in TH centers during a time of growing TH dissemination in the state of New Jersey (NJ).

**Methods:**

Using a linked database of prehospital, hospital, and mortality records for NJ in 2009-2010, we compared rates of neurologically intact survival at discharge and at 30 days for OHCA patients transported to TH centers (N = 2363) versus other hospitals (N = 2479). We used logistic regression to adjust for patient and hospital covariates. To account for potential endogeneity in prehospital transportation decisions, we used an instrumental variable (IV) based on differential distance to the nearest TH and non-TH hospitals.

**Results:**

Patients taken to TH centers were older, more likely to have a witnessed arrest, more likely to receive defibrillation, and waited a shorter amount of time for initial EMS response. Also, TH hospitals were larger, more likely to be teaching facilities, and operated in a service area with a relatively lower poverty rate compared to hospitals statewide. A Stock-Yogo test confirmed the strength of our IV (F = 2349.91, *p* < 0.0001). Nevertheless, the data showed no evidence of endogenous transportation to TH centers related to in-hospital survival (Z = -0.08, *p* = 0.934) or 30-day survival (Z = 0.94, *p* = 0.349). In logistic regression models, treatment at a TH center was associated with greater odds of 30-day neurologically intact survival (OR = 1.70; 95 % CI: 1.19 – 2.42) but not associated with the odds of neurologically intact survival to hospital discharge (OR = 0.90; 95 % CI: 0.61 – 1.31).

**Conclusions:**

Post-arrest outcomes are more favorable at TH centers but these improved outcomes are not apparent until after hospital discharge. This finding may reflect superior care by TH centers in later stages of post-arrest treatment such as care provided in the intensive care unit, which has greater potential to affect longer term outcomes than initial treatment in the emergency department.

**Electronic supplementary material:**

The online version of this article (doi:10.1186/s12913-015-1199-z) contains supplementary material, which is available to authorized users.

## Background

Every year, more than 300,000 U.S. residents experience an out-of-hospital cardiac arrest (OHCA) [1, 2]. Corresponding rates of survival to hospital discharge are very low, ranging from 1.1 % to 8.1 % across regions, and survivors often experience severe neurological impairment [3].

National guidelines have called for the use of therapeutic hypothermia (TH), which involves reducing the body’s core temperature for an extended period of time (e.g., 12-24 h), on initial survivors of OHCA [4, 5]. These guidelines are based on the results of three clinical trials demonstrating the efficacy of TH for improving rates of neurologically intact survival [6–8]. These trials were small (with a combined population of 375), conducted outside of the United States, and focused on a narrowly targeted group of patients receiving treatment for OHCA. In contrast, the real-world application of TH involves a much broader range of patient selection, cooling techniques, and other implementation details [9]. In addition, a recent trial raised questions about which temperature should be targeted after cardiac arrest [10]. Nevertheless, targeted temperature management (TTM) has come to be viewed as one of many crucial elements in the post cardiac arrest “bundle of care”, which also includes management of shock, hemodynamic management, seizure suppression, and glucose control [5, 11].

In addition to hospital-based care, OHCA patients rely on rapid intervention by prehospital emergency medical services (EMS). This intervention includes in-the-field procedures such as cardiopulmonary resuscitation (CPR) as well as the choice of destination hospital. Moreover, the choice of destination hospital is potentially endogenous in the sense that prehospital EMS personnel may decide on where to transport patients based on clinical characteristics that are unobservable to researchers but related to survival outcomes. Thus, it is not well established whether and how prehospital transportation to TH centers affects survival outcomes for OHCA patients.

To address these issues, we used a unique database of linked prehospital, hospital, and mortality records for the state of New Jersey where TH adoption by hospitals increased dramatically from 2004 to 2011 [9]. Using data from 2009-2010, we compared neurologically intact survival outcomes for patients treated at TH centers versus those treated at other hospitals. To account for potential endogeneity in prehospital transportation of patients, we used an instrumental variable (IV) approach based on distances from the scene of the OHCA to the nearest TH and non-TH facilities.

## Methods

### Study design

We conducted a retrospective analysis using statewide linked hospital, prehospital EMS, and mortality data for NJ. The study protocol was approved by the Institutional Review Boards (IRBs) at Rutgers University and the New Jersey Department of Health (DOH). Because the study was based on secondary data and the study team had no access to personal identifiers, written informed consent was not required by the IRBs.

### Setting

New Jersey is a densely populated state of 8.8 million residents [12]. There are 73 acute care hospitals operating in the state, all of which are required by state law to maintain a full-service emergency department (ED) 24 h per day. The number of hospitals adopting TH grew from none in 2004 to 38 in 2010. The state’s prehospital emergency medical services (EMS) include a mix of career and volunteer basic life support (BLS) ambulance companies. In most communities, private or municipal BLS units are supplemented by 21 hospital-based advanced life support (ALS) units staffed by career paramedics. BLS and ALS units are dispatched simultaneously to high acuity calls, including cardiac arrests. Paramedics operate under statewide protocols for cardiac arrest. After consultation with a physician, paramedics may terminate resuscitation attempts on-scene after 20-30 min of unsuccessful efforts.

### Data sources and record linkage

The study data consisted of three statewide administrative databases containing patient-level EMS, hospital utilization, and death information, which we supplemented with hospital-level survey data to identify TH centers. We obtained EMS data from the NJ EMS Data Warehouse (EMSDW), which was created as a result of a statewide EMS review mandated by the NJ state legislature and allows EMS practitioners to record all clinical and demographic data on electronic health records [13]. The EMSDW consolidates these electronic records into a single statewide data set. All ALS agencies are required to submit data to the EMSDW. While BLS agency participation is not required, approximately 50 % provide data to the EMSDW. The data captured by the EMSDW include vital signs, procedures performed, response times, resuscitation attempts, patient demographics, and patient identifiers.

The source of hospital utilization data is the New Jersey Discharge Data Collection System (NJDDCS), which contains the universe of uniform billing (UB) records for all inpatient and emergency department (ED) encounters in the state’s hospitals. Hospitals submit claims on a daily basis to the NJ DOH, which edits and standardized claims and retains them in a centralized database. The data captured by the NJDDCS include diagnoses, procedures, patient demographics, and discharge disposition.

The source of mortality data is the state vital records system maintained by the NJ DOH. Under agreements with other states, the NJ DOH obtains mortality records for NJ residents who died outside of NJ.

We linked the data sets using LinkKing© software, which employs a combination of probabilistic and deterministic linkage methods [14]. Linkage was based primarily on patient name, date of birth (DOB), and Social Security Number (SSN) with patient sex, race, ethnicity, and residential zip code as additional linking variables. Although name, DOB, and SSN are not available on public use files, they are maintained by the NJ DOH. Under a special arrangement for this project, the Department’s Center for Health Statistics implemented the required linkages using patient identifiers under state auspices, and delivered a de-identified, linked database to the study team for further preparation and analysis.

### Patients

We included adult (ages 19 and older) patients who were treated by EMS for OHCA. Using the EMSDW, we identified cardiac arrests as individuals coded as “cardiac arrest” for call type, those receiving CPR or defibrillation, and individuals with a first monitored cardiac rhythm of ventricular fibrillation (VF), ventricular tachycardia (VT), pulseless electrical activity (PEA), or asystole. We excluded from our analysis all cases where resuscitation attempts were terminated in the field, those where the EMS record could not be linked to a hospital record, and hospital transfers where patients could not be followed throughout the entire episode of care.

### Outcome measures

We examined two outcomes: (1) neurologically intact survival to hospital discharge and (2) neurologically intact 30-day survival (i.e., 30 days after the cardiac arrest). Following the approach used in the clinical trial conducted by Bernard et al. [7], we used hospital discharge codes in the NJDDCS to proxy neurological status (and mortality records to measure survival within 30 days of the arrest). Specifically, we defined neurologically intact survival as discharge/transfer to home/self-care, another hospital or short term acute care facility, rehabilitation facility, court/law enforcement, or left against medical advice. We defined poor neurological outcomes as all other discharge destinations (e.g., discharged to hospice or nursing home) and death.

### Identification of TH centers

Since the application of TH usually does not affect hospital reimbursement, this procedure is rarely recorded in hospital billing records. Thus, we were unable to utilize hospital billing records to identify individual patients who received TH. Instead, we used a prior survey of acute care hospitals in NJ to define TH centers as hospitals that adopted TH in the treatment of initial OHCA survivors [9]. Since some hospitals initiated their TH programs during the study period (2009-2010), we classified these facilities as TH centers during and after the month of implementation and as non-TH centers in the prior months. Among the state’s 73 full-service hospitals, 18 adopted TH before the study period began, 23 adopted during the study period, and 32 did not use the procedure at all.

### Analysis

We estimated the association between treatment at a TH center and the two OHCA survival outcomes using multiple logistic regression. Model covariates included incident, patient, and hospital characteristics. The incident characteristics we considered were year, whether the arrest was witnessed before EMS arrival, defibrillation attempted, shockable rhythm, response time, scene time, and transport time. The patient characteristics we considered were sex, age, race/ethnicity, and insurance coverage (defined as expected primary payer on the hospital record). The hospital characteristics we considered were number of beds, membership in the Council of Teaching Hospitals (COTH), and the poverty rate in the hospital service area (defined as the smallest number of zip codes accounting for at least 90 % of all hospital volume).

As mentioned above, patient transportation to TH centers is potentially endogenous, since prehospital EMS personnel may exercise discretion in their choice of destination hospital. In theory, prehospital EMS personnel may be more likely to take patients to TH centers when they believe these patients are good candidates for TH treatment due to patient risk factors that are unobservable in our data. If so, then ordinary/naive estimates of the relationship between transportation to a TH center and survival outcomes would be biased by unmeasured confounding [15]. To address this issue, we constructed an instrumental variable (IV) to separate patient-level variation in TH versus non-TH hospital into two components: 1) an exogenous component unrelated to likelihood of survival and 2) the remaining component, which contains unmeasured and potentially endogenous patient characteristics that may be related to likelihood of survival.

In this context, a suitable IV must be strongly related to whether a patient is transported to a TH center but have no direct relationship to OHCA survival [16]. In other words, the IV affects survival only through its association with transport to a TH center. For our analysis, we used an IV defined as the differential distance between the closest TH and non-TH hospitals. The IV calculation was based on the distance between the geographic centroid of the EMS incident zip code and the street addresses of the nearest TH and non-TH hospitals.

Since we had exactly one IV to predict exactly one potentially endogenous treatment variable, our data did not satisfy the over-identification condition required to formally test whether our differential distance variable is exogenous (i.e., not directly related to survival outcomes) [15]. Nevertheless, distance-to-hospital variables such as ours are often used as IVs on the grounds that direct association with patient outcomes is generally considered implausible [17–19].

We implemented our IV strategy using the 2-stage residual inclusion method developed by Terza et al. [20], which extends previously developed IV estimation for linear models to non-linear models such as logistic regression. In stage 1, we used ordinary linear regression to predict transport to a TH center based on the IV and all other exogenous independent variables listed above. In stage 2, we estimated logistic regression models to predict each survival outcome (i.e., in-hospital and 30-day) on the basis of transport to a TH facility, the residual from stage 1, and the exogenous independent variables. After controlling for these factors, the coefficient for the TH variable provides a consistent estimate of the relationship between TH transport and survival outcomes by Terza et al. [20].

Additionally, the coefficient for the stage 1 residual in the stage 2 model provides a test of the endogeneity of transportation to a TH center. If this coefficient is significantly different from 0, endogeneity is confirmed. Otherwise, more efficient and consistent estimates are generated from logistic regression without the stage 1 residual [15, 20].

We used the Stock-Yogo F-test to confirm that our IV is sufficiently strong to reliably account for unmeasured confounding [16]. We also used likelihood ratio tests to choose between ordinary logistic regression and mixed effects logistic regression with hospitals specified as random clustering units. All analyses were performed using STATA 13.0.

## Results

**Fig. 1:**
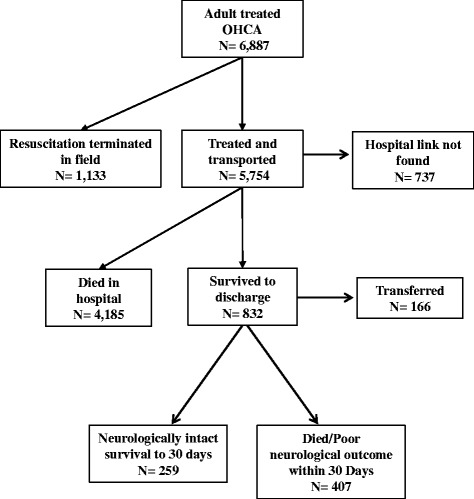
Outcomes for out-of-hospital cardiac arrest cases

We identified 6887 adults treated by EMS for OHCA in 2009-2010 (Fig. 1). Of these, 1133 cases had resuscitation efforts terminated in the field and were removed from the analysis. Among the 5754 treated and transported cases, 5017 (87.2 %) could be linked reliably to hospital and mortality records, while the remaining 737 cases contained insufficient information for reliable linkage. Among the 5107 linked cases, 166 were transferred from the initial destination hospital to other hospitals that were not identified on the NJDDCS record. Since these cases could not be followed throughout the entire course of OHCA treatment, they were excluded from the analysis. (Among these 166 excluded cases, 46.4 % were initially transported to a TH center, a percentage that is very similar to the corresponding percentage in the final study sample documented below.) After excluding 9 additional cases with missing values of covariates, we retained 4842 observations in the analysis below.

Almost half (48.8 %) of the OHCA patients in our analysis were transported to TH centers (Table 1). These patients experienced significantly higher rates of neurologically intact survival to discharge (11.9 % at TH centers versus 8.2 % at other hospitals) and neurologically intact 30-day survival (7.4 % at TH centers and 3.4 % at other hospitals). Patients taken to TH centers also were older, more likely to have a witnessed arrest, more likely to receive defibrillation, and waited a shorter amount of time for initial EMS response. In addition, these patients were more likely to receive care from hospitals that were larger, teaching facilities, and operated in a service area with a lower poverty rate. There were more patients treated at TH centers in 2010 than in 2009.

**Table 1:** Characteristics of out-of-hospital cardiac arrest patients treated at therapeutic hypothermia (TH) centers versus other hospitals

Variable^b^	Patients treated at TH centers	Patients treated at other hospitals
	(N = 2,363)	(N = 2,479)
Survival to discharge with normal neurological status	11.9 %	8.2 %^a^
30-day survival with normal neurological status	7.4 %	3.4 %^a^
Year		
2009	33.6 %	48.4 %^a^
2010	66.4 %	51.6 %
Witnessed arrest	60.0 %	55.0 %^a^
Bystander CPR	6.5 %	7.6 %
Defibrillation by EMS	52.3 %	46.1 %^a^
Shockable rhythm^c^	9.2 %	8.7 %
EMS response time (dispatch to arrival on-scene)		
Less than 4 minutes	16.8 %	14.4 %^a^
4-8 minutes	41.6 %	36.0 %
More than 8 minutes	41.6 %	49.6 %
EMS time on scene		
Less than 4 minutes	1.7 %	1.3 %
4-8 minutes	6.1 %	7.1 %
More than 8 minutes	92.3 %	91.7 %
EMS transport time (departure from scene to hospital arrival)		
Less than 4 minutes	22.5 %	20.9 %
4-8 minutes	32.6 %	34.9 %
More than 8 minutes	44.9 %	44.2 %
Female sex	37.2 %	36.4 %
Age in years		
Less than 50	15.5 %	17.5 %^a^
50-65	28.1 %	29.9 %
66-80	30.0 %	30.3 %
81 and above	26.5 %	22.3 %
Race/ethnicity		
White	67.6 %	65.4 %
Black	17.3 %	18.4 %
Hispanic	6.4 %	7.8 %
Asian/Pacific Islander	2.9 %	2.6 %
Other non-white	5.8 %	5.8 %
Expected primary payer		
Medicare	55.3 %	52.6 %
Medicaid	3.3 %	4.4 %
Private	25.0 %	25.5 %
Self-pay/uninsured	13.9 %	15.5 %
Other	2.5 %	2.1 %
Number of hospital beds		
Less than 200	19.6 %	46.1 %^a^
200-399	49.8 %	42.4 %
400 and above	30.6 %	11.5 %
Teaching hospital	14.8 %	7.6 %^a^
Poverty rate in hospital service area	6.6 %	7.1 %^a^

^a^Difference between patients at therapeutic hypothermia (TH) centers and other hospitals is statistically significant at the 1 % level. All other differences are not statistically significant at the 5 % level

^b^Differences between patients at TH centers versus other hospitals were tested using Chi-square tests except for poverty rate in hospital service area where a *t* test was used

^c^Shockable rhythm was identified as ventricular fibrillation (VF), ventricular tachycardia (VT), or unknown AED shockable rhythm. Non-shockable rhythms were asystole and pulseless electrical activity

The Stock-Yogo test confirmed the use of differential distance as an appropriate IV (F = 2349.91, *p* < 0.0001). The stage 1 residual was statistically insignificant in both stage 2 models (In-hospital survival: Z = -0.08, *p* = 0.934; 30-day survival: Z = 0.94, *p* = 0.349). As a result, our data provided no evidence of endogenous transportation to TH centers (see Tables, Additional file 1, for full stage 1 and stage 2 estimation results). Thus, we focus on estimates from non-IV based logistic regression models. In both of these models, likelihood ratio tests rejected the ordinary logistic regression specification in favor of the mixed effects model for both study outcomes (Survival to discharge: Chi-square = 143.67, *p* < 0.01; 30-day survival: Chi-square = 11.86, *p* < 0.01).

After estimating several model specifications, we produced the final specifications shown in Table 2. Final specifications exclude covariates listed in Table 1 that were never jointly or individually significant in any of the specifications considered. Moreover, inclusion of these “additional” covariates did not fundamentally change the observed relationships between transportation to a TH hospital and study outcomes.

**Table 2:** Odds ratios from logistic regression analysis

	Odds ratio^a^	95 % Confidence interval^a^	Odds ratio^b^	95 % Confidence interval^b^
Treated at TH center	0.90	0.61, 1.31	1.70	1.19, 2.42
Year 2010	0.83	0.67, 1.03	0.81	0.61, 1.06
Witnessed arrest	1.44	1.17, 1.79	1.77	1.32, 2.38
Defibrillation by EMS	1.31	1.06, 1.62	1.54	1.16, 2.04
Shockable rhythm	1.15	0.82, 1.59	1.34	0.91, 1.97
Response time (dispatch to arrival on-scene)				
(reference: less than 4 minutes)				
4-8 minutes	0.98	0.73, 1.31	0.56	0.39, 0.79
More than 8 minutes	0.88	0.65, 1.18	0.50	0.35, 0.70
Female sex (reference: male sex)	0.91	0.74, 1.13	0.89	0.66, 1.19
Age in years (reference: less than 50)				
50-65	0.53	0.40, 0.70	0.61	0.43, 0.86
66-80	0.52	0.37, 0.74	0.49	0.32, 0.76
81 and above	0.41	0.28, 0.61	0.19	0.11, 0.34
Race/ethnicity (reference: White)				
Black	0.92	0.69, 1.22	0.83	0.57, 1.21
Hispanic	0.92	0.61, 1.39	0.77	0.43, 1.37
Asian/Pacific Islander	1.31	0.77, 2.22	0.80	0.34, 1.90
Other non-white	1.41	0.94, 2.13	1.24	0.74, 2.08
Expected primary payer (reference: Medicare)				
Medicaid	1.37	0.83, 2.27	0.81	0.37, 1.75
Private	1.30	0.97, 1.75	1.38	0.95, 2.01
Self-pay/uninsured	0.89	0.62, 1.28	0.61	0.37, 1.00
Other	1.09	0.54, 2.19	1.57	0.72, 3.41
Number of hospital beds (reference: less than 200)				
200-399	2.19	1.24, 3.88	1.54	0.98, 2.41
400 and above	2.83	1.33, 6.05	3.32	1.98, 5.59

^a^Dependent variable is neurologically intact survival to hospital discharge

^b^Dependent variable is 30-day neurologically intact survival

After adjusting for covariates, treatment at a TH center was not significantly associated with neurologically intact survival to hospital discharge. However, after adjusting for confounders, the odds of 30-day neurologically intact survival were 70 % greater for patients taken to TH centers relative to other patients (Table 2). Findings were very similar in sensitivity analyses that examined raw survival outcomes without accounting for neurological status (see Appendix for details).

## Discussion

In this study, we found a strong positive association between transportation to a TH center and neurologically intact 30-day survival among OHCA patients. We did not, however, observe a similar relationship between TH centers and neurologically intact survival to hospital discharge. In interpreting these results, one must bear in mind that our designation of a “TH center” was an indicator of each hospital’s capabilities, not a measure of whether TH was provided to individual patients.

This distinction is important, since some TH centers provide the procedure to only a very narrowly selected group of patients on the basis of ECG rhythms, age, and other selection criteria [9]. Thus, our analysis evaluates the effectiveness of care rendered to all OHCA patients at hospitals that have adopted TH, regardless of whether any TTM was actually performed on each individual.

Recently established consensus guidelines emphasize that optimal post-arrest care entails a broad bundle of key elements in addition to TTM, including appropriate management of shock, hemodynamic management, seizure suppression, and glucose control [5]. Our findings suggest that hospitals providing TH may be more attuned to this broad set of care elements for all of their OHCA patients.

Another important observation is that although 30-day neurologically intact survival was associated with transportation to a TH center, there was no difference in survival to hospital discharge. This finding is similar to prior research using data from the Resuscitation Outcomes Consortium (ROC), which demonstrated that 24-h OHCA survival in the community often differs from conditional survival to hospital discharge [21]. Both of these related findings suggest that immediate and longer-term outcomes may be influenced by different mechanisms. For example, our findings may reflect superior care by TH centers in later stages of post-arrest treatment such as care provided in the intensive care unit, which has greater potential to affect longer term outcomes than initial treatment in the emergency department. In addition, longer-term survival may also be influenced by well-coordinated post-discharge follow-up care. Unfortunately, we are unable to ascertain from our database whether the improved longer-term survival outcomes are driven by interventions taking place within TH centers or during the post-discharge follow-up stage.

Our study provides unique and important insights into the “real-world” consequences of TH adoption. Previous work has been limited to small-scale clinical trials outside of the U.S. (where acute and post-acute care delivery systems are often organized and operated very differently) and observational studies using before-after designs to describe care at single TH centers [6–8, 22–24]. In contrast, our large-scale study of NJ includes a much larger number of patients and a heterogeneous range of TH and non-TH hospitals. In addition, our analysis is the first to our knowledge that uses an IV approach to account for the potential endogeneity of patient transportation to TH centers by prehospital EMS. Our finding that transportation to a TH center is not endogenous suggests that local EMS units do not alter the transport destination of OHCA patients on the basis of survival probability or likely benefit from care at a TH center.

Some experts have argued in favor of regional systems of care for OHCA, similar to those established for trauma, burn, stroke, and STEMI centers [25]. Specifically, if a patient in cardiac arrest is resuscitated in the field by EMS, then the arrest outcome may be improved if the patient is taken directly to a TH center, even if this means bypassing a closer non-TH hospital. Our finding of more favorable outcomes at TH centers provides support for regionalization. Moreover, even though longer EMS response time was independently associated with worse outcomes, we found that EMS transport time to the destination hospital was not a significant predictor of neurologically intact survival. Our analysis is also consistent with prior work showing better OHCA outcomes in larger hospitals but no significant difference between teaching and non-teaching hospitals [26].

Our findings should be viewed in light of some limitations. Since we were unable to identify individual patients who received TTM, our analysis is an evaluation of the care provided by TH centers and not an evaluation of TH itself. Similarly, we did not have information on parallel resuscitation interventions such as use of percutaneous coronary intervention (PCI), which is known to affect OHCA outcomes but is not used uniformly across hospitals [27–31]. We were, therefore, unable to determine whether favorable 30-day outcomes at TH centers were influenced by use of PCI or other processes of care. Our data also did not allow us to identify the elapsed time between arrest and return of spontaneous circulation, which is strongly associated with brain injury and overall survival.

Because detailed neurological assessments were are not available in our hospital data, we had to proxy neurological status among OHCA survivors using hospital discharge codes. More detailed neurological assessments would likely change the outcome classification for some patients in the study but we do not expect the classification would change differently for patients transported to TH centers versus other hospitals. Similarly, some OHCA patients may have originally been nursing home residents who would have returned to their nursing home if they survived to hospital discharge. Such individuals would be classified as having a poor outcome even though they may have returned to their original neurological status after hospital discharge. Although our data do not allow us to identify how often these situations occur, the imprecision in our measure of neurological status does not create any systematic bias for or against TH centers.

In addition, sensitivity analyses confirm our main findings based on the proxy measure for neurological status. First, although post-discharge placement may be influenced by socioeconomic and geographic concerns, our findings were not influenced by patient insurance status or poverty rates in hospital service areas. Second, conclusions were identical in models that examined survival outcomes regardless of neurological status.

Although participation in New Jersey’s EMS data collection system is nearly universal among ALS units, responses to OHCA calls from a number of BLS units are likely to be missing. Nevertheless, we expect these missing cases to be minimal, since most responses to OHCA will involve ALS response at some point of the episode.

Finally, despite our use of validated data linkage software [32], the records for 737 patients treated and transported by EMS were lost due to insufficient identifying information for reliable linkage. Records for an additional 166 patients were lost after these individuals were transferred to other hospitals. It is also possible that some records may have been linked erroneously. While the existence of missing or erroneous links adds uncertainty to our conclusions, we do not expect these issues to add any systematic bias for or against the outcomes measured at TH centers. In addition, confidence in the validity of our study data is enhanced by its comparability to the previously published national rate of survival to hospital discharge among adults treated by EMS for OHCA: 13.7 % for NJ in 2009-2010 versus 9.8 % nationwide in 2010 [2].

## Conclusion

Despite these caveats, our study provides evidence that post-arrest outcomes are more favorable when patients are transported to TH centers. These improved outcomes are not apparent, however, until after hospital discharge. This finding may reflect superior care by TH centers in later stages of post-arrest treatment such as care provided in the intensive care unit, which has greater potential to affect longer term outcomes than initial treatment in the emergency department. More research is needed to delineate specifically the mechanisms that lead to these improved outcomes at later stages of post-arrest treatment.

## Additional file

Additional file 1:
**Instrumental variable estimates.** Tables for full stage 1 and stage 2 instrumental variable estimation results (PDF 244 kb)

## Abbreviations

ALS: Advanced life support
BLS: Basic life support
COTH: Council of teaching hospitals
CPR: Cardiopulmonary resuscitation
DOB: Date of birth
DOH: Department of health
ED: Emergency department
EMS: Emergency medical services
EMSDW: Emergency medical services data warehouse
IRB: Institutional review board
IV: Instrumental variable
NJ: New Jersey
NJDDCS: New Jersey discharge data collection system
OHCA: Out-of-hospital cardiac arrest
PEA: Pulseless electrical activity
ROC: Resuscitation outcomes consortium
SSN: Social security number
TH: Therapeutic hypothermia
TTM: Targeted temperature management
UB: Uniform billing
VF: Ventricular fibrillation
VT: Ventricular tachycardia
