# Supplementary material for: Prehospital transportation to therapeutic hypothermia centers and survival from out-of-hospital cardiac arrest
Source: BMC Health Serv Res. 2015 Dec 2;15:533. doi: 10.1186/s12913-015-1199-z (PMC4668679; doi:10.1186/s12913-015-1199-z)
Supplement: Additional file 1: — Instrumental variable estimates. Tables for full stage 1 and stage 2 instrumental variable estimation results (PDF 244 kb) [file 12913_2015_1199_MOESM1_ESM.pdf]

Supplemental digital content for
"Prehospital transportation to therapeutic hypothermia centers and survival from out-of-hospital cardiac arrest"

Table 1 First stage model predicting transport to therapeutic hypothermia (TH) center

	Coefficient	Standard Error	p-value	95% CI*	
Instrumental variable: Differential distance	-0.037	0.001	0.000	-0.039	-0.036
Year 2010	0.041	0.011	0.000	0.018	0.063
Witnessed arrest	0.013	0.011	0.261	-0.009	0.035
Defibrillation by EMS	0.034	0.012	0.003	0.011	0.057
Shockable rhythm	-0.009	0.020	0.642	-0.049	0.030
Response time (Dispatch to arrival on-scene) (Reference: Less than 4 minutes)					
4-8 minutes	0.000	0.017	0.981	-0.032	0.033
More than 8 minutes	0.000	0.016	0.998	-0.032	0.032
Female sex (Reference: Male sex)	-0.002	0.012	0.842	-0.025	0.021
Age in years (Reference: Less than 50)					
50-65	-0.006	0.017	0.747	-0.039	0.028
66-80	-0.024	0.020	0.243	-0.063	0.016
81 and above	-0.002	0.022	0.934	-0.044	0.041
Race/ethnicity (Reference: White)					
Black	-0.052	0.015	0.001	-0.082	-0.022
Hispanic	-0.087	0.022	0.000	-0.130	-0.044
Asian/Pacific Islander	-0.070	0.034	0.041	-0.136	-0.003
Other non-white	-0.066	0.024	0.006	-0.113	-0.019
Expected primary payer (Reference: Medicare)					
Medicaid	-0.008	0.031	0.808	-0.069	0.054
Private	-0.022	0.016	0.170	-0.055	0.010
Self-pay/uninsured	-0.037	0.020	0.059	-0.076	0.001
Other	0.043	0.038	0.259	-0.032	0.119
Number of hospital beds (Reference: Less than 200)					
200-399	0.110	0.013	0.000	0.084	0.135

	Coefficient	Standard Error	p-value	95% CI[*]	
400 and above	0.284	0.016	0.000	0.254	0.315
Constant	0.451	0.027	0.000	0.397	0.504
R^2=0.421					
F(21, 4795)=165.73					
p<0.001					
Staiger-Stock test					
F(1, 4795)=2349.91					
p<0.001					

[*]Confidence interval.

Table 2 Second stage models predicting survival outcomes adjusting for unobservable factors associated with patient transport to therapeutic hypothermia (TH) centers

	**Neurologically Intact Survival to Hospital Discharge**			**30-day Neurologically Intact Survival**		
	**Odds Ratio**	**95% CI***		**Odds Ratio**	**95% CI***	
Stage 1 residual	0.975	0.529	1.795	1.382	0.702	2.724
Treated at TH center	0.913	0.519	1.607	1.350	0.751	2.428
Year 2010	0.831	0.668	1.035	0.836	0.631	1.108
Witnessed arrest	1.443	1.167	1.785	1.779	1.328	2.383
Defibrillation by EMS	1.312	1.061	1.622	1.548	1.164	2.057
Shockable rhythm	1.147	0.824	1.596	1.331	0.903	1.962
Response time (Dispatch to arrival on-scene) (Reference: Less than 4 minutes)						
4-8 minutes	0.978	0.730	1.309	0.558	0.393	0.792
More than 8 minutes	0.879	0.655	1.180	0.494	0.347	0.702
Female sex (Reference: Male sex)	0.912	0.735	1.133	0.887	0.663	1.187
Age in years (Reference: Less than 50)						
50-65	0.529	0.400	0.700	0.605	0.428	0.858
66-80	0.525	0.373	0.738	0.490	0.316	0.759
81 and above	0.402	0.272	0.595	0.192	0.108	0.343
Race/ethnicity (Reference: White)						
Black	0.918	0.690	1.223	0.836	0.574	1.217
Hispanic	0.920	0.607	1.396	0.745	0.416	1.333
Asian/Pacific Islander	1.305	0.767	2.220	0.796	0.336	1.886
Other non-white	1.406	0.930	2.124	1.212	0.720	2.042
Expected primary payer (Reference: Medicare)						
Medicaid	1.378	0.831	2.285	0.807	0.373	1.747
Private	1.305	0.970	1.754	1.376	0.948	1.998
Self-pay/uninsured	0.899	0.626	1.291	0.602	0.364	0.995
Other	1.099	0.546	2.212	1.592	0.729	3.476
Number of hospital beds (Reference: Less than 200)						

	Neurologically Intact Survival to Hospital Discharge			30-day Neurologically Intact Survival		
	**Odds Ratio**	**95% CI***		**Odds Ratio**	**95% CI***	
200-399	2.169	1.216	3.869	1.618	1.024	2.556
400 and above	2.812	1.290	6.133	3.633	2.093	6.308
Likelihood ratio test for mixed effects vs. ordinary logistic regression						
Chi-square statistic	139.78			11.48		
p-value	<0.001			<0.001		

*Confidence interval.

Table 3 Odds ratios from logistic regression models predicting survival regardless of neurological status

	Odds ratio*	95% Confidence Interval*	Odds ratio†	95% Confidence Interval†
Treated at TH center	0.83	0.59, 1.15	1.54	1.11, 2.13
Year 2010	0.84	0.70, 1.01	0.79	0.63, 1.00
Witnessed arrest	1.59	1.32, 1.92	1.85	1.45, 2.36
Defibrillation by EMS	1.14	0.95, 1.37	1.28	1.01, 1.62
Shockable rhythm	1.39	1.05, 1.84	1.56	1.12, 2.16
Response time (dispatch to arrival on-scene) (reference: less than 4 minutes)				
4-8 minutes	0.89	0.69, 1.14	0.61	0.45, 0.82
More than 8 minutes	0.77	0.60, 0.99	0.51	0.38, 0.69
Female sex (reference: male sex)	1.07	0.89, 1.29	1.03	0.81, 1.31
Age in years (reference: less than 50)				
50-65	0.62	0.48, 0.80	0.69	0.50, 0.94
66-80	0.63	0.46, 0.85	0.58	0.40, 0.85
81 and above	0.50	0.35, 0.70	0.30	0.19, 0.47
Race/ethnicity (reference: White)				
Black	0.85	0.66, 1.09	0.78	0.56, 1.08
Hispanic	1.11	0.79, 1.57	1.23	0.80, 1.88
Asian/Pacific Islander	1.20	0.74, 1.95	0.74	0.35, 1.58
Other non-white	1.41	0.98, 2.03	1.21	0.77, 1.91
Expected primary payer (reference: Medicare)				
Medicaid	1.75	1.14, 2.67	0.96	0.53, 1.74
Private	1.08	0.84, 1.40	1.10	0.80, 1.51
Self-pay/uninsured	0.73	0.53, 1.00	0.45	0.29, 0.70
Other	0.77	0.40, 1.48	1.05	0.51, 2.17
Number of hospital beds (reference: less than 200)				
200-399	1.70	1.06, 2.73	0.27	0.86, 1.95
400 and above	2.55	1.36, 4.81	0.73	1.72, 4.71

* Dependent variable is survival to hospital discharge.

† Dependent variable is 30-day survival.
